# Evaluating the Usability of an HIV Prevention Artificial Intelligence Chatbot in Malaysia: National Observational Study

**DOI:** 10.2196/70034

**Published:** 2025-07-15

**Authors:** Zhao Ni, Sunyoung Oh, Rumana Saifi, Iskandar Azwa, Frederick L Altice

**Affiliations:** 1 School of Nursing, Yale University, 400 West Campus Drive, Orange, CT, 06477-3646, United States, 1 2037373039; 2 Center for Interdisciplinary Research on AIDS (CIRA), Yale University, New Haven, CT, United States; 3 Centre of Excellence for Research in Infectious Diseases & AIDS (CERiA), Universiti Malaya, Kuala Lumpur, Malaysia; 4 Department of Social and Preventive Medicine, Universiti Malaya, Kuala Lumpur, Malaysia; 5 Infectious Disease Unit, Faculty of Medicine, Universiti Malaya, Kuala Lumpur, Malaysia; 6 Section of Infectious Disease, Department of Internal Medicine, Yale School of Medicine, New Haven, CT, United States; 7 Division of Epidemiology of Microbial Diseases, Yale School of Public Health, New Haven, CT, United States

**Keywords:** artificial intelligence, chatbot, feasibility, HIV prevention, HIV testing, men who have sex with men, MSM, mHealth, pre-exposure prophylaxis, PrEP, usability, mobile health

## Abstract

**Background:**

Malaysia, an upper middle-income country in the Asia-Pacific region, has an HIV epidemic that has transitioned from needle sharing to sexual transmission, mainly in men who have sex with men (MSM). MSM are the most vulnerable population for HIV in Malaysia. In 2022, our team developed a web-based artificial intelligence (AI) chatbot and tested its feasibility and acceptability among MSM in Malaysia to promote HIV testing. To enhance the usability of the AI chatbot, we made it accessible to the public through the website called MYHIV365 and tested it in an observational study.

**Objective:**

This study aimed to test the usability of an AI chatbot in promoting HIV testing among MSM living in Malaysia.

**Methods:**

This observational study was conducted from August 2023 to March 2024 among 334 MSM. Participants were recruited through community outreach and social-networking apps using flyers. The interactions between participants and the AI chatbot were documented and retrieved from the chatbot developer’s platform. Data were analyzed following a predefined metrics using R software (Posit Software, PBC).

**Results:**

The AI chatbot interacted with 334 participants, assisting them in receiving free HIV self-testing kits, offering information on HIV, pre-exposure prophylaxis (PrEP), and mental health, and providing details of 220 MSM-friendly clinics, including their addresses, phone numbers, and operating hours. After the study, 393 human-chatbot interactions were documented on the chatbot developer’s platform. Most participants (304/334, 91.0%) interacted with the AI chatbot once, 30 (9.0%) engaged 2 or more times at different intervals. Participants’ interaction time with the chatbot varied, ranging from 1 to 31 minutes. The AI chatbot properly addressed most participants’ questions (362/393, 92.1%) about HIV and PrEP. However, in 31 interactions, participants posed additional questions to the chatbot that were not programmed into the chatbot algorithms, resulting in unanswered interactions.

**Conclusions:**

The web-based AI chatbot demonstrated high usability in delivering HIV self-testing kits and providing clinical information on HIV testing, PrEP, and mental health services. To enhance its usability in community and clinical settings, the chatbot must offer personalized health information and precise interaction, powered by sophisticated machine learning algorithms. In addition, establishing an effective connection between the AI chatbot and health care systems to eliminate stigma and discrimination toward MSM is crucial for the future implementation of AI chatbots.

## Introduction

Men who have sex with men (MSM) are the most vulnerable population for HIV globally, accounting for 44% of new HIV cases in the Asia-Pacific region [1]. Malaysia, an upper middle-income country in this region, is experiencing one of the fastest growing HIV epidemics in MSM [2,3], characterized by a shift from needle sharing to sexual transmission [4]. The vulnerability of MSM to HIV is associated with various factors, including the stigma and discrimination against HIV and same-sex sexual behaviors [3,5,6]. In Malaysia, same-sex sexual behaviors are criminalized by both secular and Shariah law [3,7], and discrimination against MSM has extended to clinical settings in physicians and medical students [5]. As a result, MSM in Malaysia must navigate through individual, clinical, and policy barriers that perpetuate stigma and discrimination to minimally access HIV-prevention services, including HIV testing and pre-exposure prophylaxis (PrEP). To increase access to quality HIV-prevention services where stigma and discrimination against MSM persist, leveraging cutting-edge technologies is crucial for developing culturally tailored interventions. Therefore, we propose using an artificial intelligence (AI) chatbot to promote HIV testing and PrEP uptake in Malaysia, aiming to counteract the discriminatory environment MSM face and increase their likelihood of accessing high-quality health care.

An AI chatbot is a software technology embedded with machine learning algorithms that can simulate human conversations through text or voice interactions [8]. Since the emergence of ChatGPT (OpenAI Inc), the world has witnessed the popularity of AI chatbot use. In the literature, numerous studies have discussed the potential of AI chatbots in promoting the efficiency of health care and improving people’s access to quality health care services [8]. In the health care industry, however, evidence of testing AI chatbots is urgently needed for guiding the future research and implementation of AI chatbot technology to complement current health care systems, particularly in countries and regions where certain vulnerable populations have limited access to high-quality nonstigmatizing health care services. Studying innovative technologies, such as AI, machine learning, and chatbots, is essential because a major barrier to the implementation of conventional digital health interventions in community and clinical settings is that they often require high-intensity and sustained human inputs, management, and maintenance [8-10]. As AI technology advances, AI chatbot–powered digital interventions are poised to overcome the barrier as they simplify the process of obtaining responses by eliminating the need for users to manually sift through search results as required in web-based searches [8] or navigating complex app interfaces among various app features. With advancements in the AI chatbot algorithms, the search for health information is likely to evolve into a streamlined Q&A process in the future.

AI chatbots applied in health care, however, must be sophisticated enough to provide precise and personalized information and update their responses based on users’ inputs without human interventions. This feature is particularly important and promising in intensively busy health care systems, where a significant amount of human labor has traditionally been required to provide health care services regularly and manually. The development and implementation of AI chatbots in health care is still in its early stages. Our team has been conducting research on AI chatbots since 2020, and we have created an AI chatbot and tested its feasibility and acceptability for promoting HIV testing among MSM in Malaysia. We found that such an AI chatbot is acceptable by MSM [3,6]. To further enhance the algorithms and features of the AI chatbot, it is essential to test its usability among a large number of end-users using predefined metrics. Therefore, we conducted this observational study to evaluate the usability of our AI chatbot for promoting HIV testing among MSM in Malaysia.

## Methods

### Study Design and Participants

The AI chatbot was created and embedded into the website MYHIV365.com. The description of the chatbot and the website has been published elsewhere [6]. In this study, participants were recruited from August 28, 2023, to March 9, 2024, through community outreach and social-networking apps. Three research assistants in Kuala Lumpur, Malaysia, distributed recruitment flyers to MSM-friendly clinics and nonprofit organizations. They also posted the flyers on social-networking apps commonly used by MSM in Malaysia, including WhatsApp, Twitter, Telegram, Facebook, TikTok, Grindr, and Instagram. The eligibility criteria for participants include: (1) aged 18 years or older, (2) able to speak and read Bahasa Malaysia or English, and (3) self-identified as MSM. Participants who reside outside Malaysia were excluded because this study aimed to create an AI chatbot that is culturally tailored for MSM living in Malaysia. We recruited 334 participants, who generated 393 interactions with the AI chatbot. An interaction was defined as a contact initiated by a user with the chatbot.

The 3 research assistants collected research data on human-chatbot interactions through the AI chatbot developer’s platform using predefined metrics (Table 1), including satisfaction, recommendation score, number of speech bubbles in each interaction, unanswered questions, interaction time, topic selection, HIV self-testing kit order, depression screening, and clinic searching. A speech bubble was defined as a unit of conversation, which is a visual element commonly used in chat interfaces to represent a chatbot’s or user’s inputs. For instance, if a user accessed the AI chatbot once and asked 10 questions, then there would be 1 interaction and 10 speech bubbles.

**Table 1.** The chatbot usability metrics.

Metrics	Description	Data type
Satisfaction with the chatbot		
Satisfaction level	Do you think this chatbot is useful? “Thumb up” or “Thumb down”	Ordinal
Frequency of the satisfaction evaluation	The frequency with which the chatbot evaluates participants’ satisfaction level.	Categorical
Recommendation score	“How likely are you to recommend this chatbot to others?” On the scale, 0 stands for extremely unlikely, and 10 stands for extremely likely.	Numerical (discrete)
Number of speech bubbles	A speech bubble is a visual element commonly used in chat interfaces to represent a chatbot’s or user’s inputs. Higher counts of speech bubbles reflect greater levels of engagement.	Numerical(discrete)
Unanswered questions		
Additional questions asked by participants	Additional questions asked by participants that were not included in the chatbot algorithms.	String (text data)
Frequency of unanswered questions	Participant’s questions to which the chatbot responded with “I cannot understand your question.”	Categorical
Interaction time		
Duration	Total time duration of a human-chatbot interaction.	Numerical (discrete)
Contact frequency	Total number of contacts from each participant. Higher frequencies reflect greater engagement with the chatbot.	Categorical
Topic selection	The frequency of topic choices (ie, HIV testing, PrEP, and mental health) selected by participants.	Categorical
HIV self-testing kit order	The number of participants who ordered an HIV self-testing kit.	Numerical (discrete)
Depression screening	The frequency of screening depression using the chatbot.	Categorical
Clinic searching	The frequency of searching for details of MSM-friendly clinics, including their addresses, phone numbers, and operating hours.	Categorical

### Statistical Analyses

Descriptive statistics were calculated to summarize the variables listed in the chatbot usability metrics. Categorical variables, such as language used, satisfaction level, satisfaction evaluation, unanswered questions, contact frequency, topic selection, depression screening, and clinic searching were reported as frequency (n) and percentage (%). Numerical variables including recommendation score, number of speech bubbles, duration, and HIV self-testing kit order were reported as mean and SD. All analyses were performed using R (version 4.3.3) software (Posit Software, PBC).

### Ethical Considerations

This study received ethical approval from the Yale University Institutional Review Board (Protocol ID: 2000030718) and the Medical Research Ethics Committee of University of Malaya Medical Centre. Participation was voluntary and data were anonymized, thus no compensations were provided. Prior to participation, all participants were provided with a Participant Information Sheet detailing the study. Participants provided electronic consent before beginning the study.

## Results

**Figure 1.**
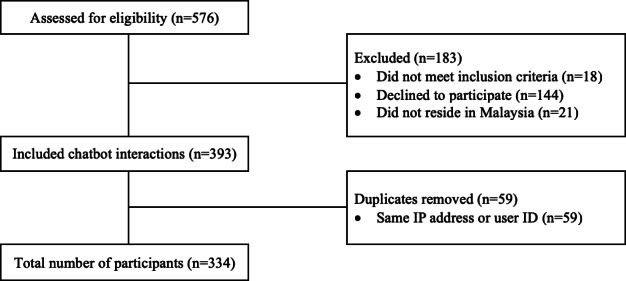
The flowchart of participants' recruitment.

As mentioned, we recruited 334 participants for this study (Figure 1).

### Language

In this study, 52.2% (205/393) of the interactions were in English, 47.1% (185/393) were in Bahasa Malaysia, and 0.8% (3/393) were in both languages.

### Participants’ Satisfaction

The AI chatbot automatically and randomly assessed participants’ satisfaction in 127 (32.3%) human-chatbot interactions out of a total of 393. The frequency of evaluations during a single interaction varied randomly; there were occasions when the chatbot assessed participants’ satisfaction multiple times but on different topics (eg, HIV testing, PrEP, and mental health). Specifically, the frequency of satisfaction evaluations about the usefulness of the information on HIV testing, PrEP, and mental health was 53, 52, and 32, respectively. We calculated all participants’ chatbot evaluations using a scoring system where thumbs up were assigned a value of +1 and thumbs down were assigned a value of −1, resulting in a mean satisfaction score of 1.3 (SD 1.1) (Table 2). Out of the 127 participants who evaluated the AI chatbot, only 14 (11%) provided negative “Thumbs down” feedback. The chatbot randomly assessed the recommendation scores of 43 participants (10.9%) using the Net Promoter Scale, yielding a mean score of 8.4 (SD 2.3).

**Table 2.** A summary of the human-chatbot interactions.

Variables	Interactions (N=393)	
	Value, n (%)	Mean (SD)
Language		—^a^
English	205 (52.2)	
Bahasa Malaysia	185 (47.1)	
Both	3 (0.8)	
Satisfaction level^b^	200 (100)	1.3 (1.1)
Thumb up	185 (92.5)	
Thumb down	15 (7.5)	
Frequency of satisfaction evaluation^c^	393	—
0	266 (67.7)	
1	77 (19.6)	
2	38 (9.7)	
3	7 (1.8)	
4	3 (0.8)	
6	1 (0.2)	
8	1 (0.2)	
Recommendation score^d^	43 (10.9)	8.4 (2.3)
Number of speech bubbles	14,262 (100)	
Chatbot	9665 (67.8)	24.6 (14.7)
Participants	4597 (32.2)	11.7 (8.4)
Frequency of unanswered questions	31 (8%)	—
1	25 (6.4)	
2	6 (1.6)	
Duration (minutes)		—
< 1	21 (5.3)	
1-5	303 (77.1)	
6-10	42 (10.7)	
>10	27 (6.9)	
Contact frequency^e^		—
1	304 (91.0)	
2	24 (7.2)	
3	4 (1.2)	
4	2 (0.6)	
Topic selection^f^		—
HIV testing	313 (79.6)	
PrEP^g^	101 (25.7)	
Mental health	49 (12.5)	
HIV self-testing kit order	260 (66.2)	—
Depression screening (PHQ-9)^h^	27 (6.9)	13.4 (7.8)
Clinic searching	93 (23.7)	—
HIV testing	25 (5.9)	
PrEP	65 (16.5)	
Mental health	16 (4.1)	

a Not applicable.

b In the scoring system, thumb up was assigned as +1, and thumb down was -1.

c The chatbot randomly evaluated 127 participants’ satisfaction score. In every evaluation, the chatbot may randomly assess more than one topic selected from HIV testing, PrEP, and mental health.

d The chatbot randomly evaluated 43 participants’ recommendation score.

e The contact frequency of 334 participants.

f Among the 393 interactions, some were related to more than one topic.

g PrEP: pre-exposure prophylaxis.

h PHQ-9: Patient Health Questionnaire-9.

### Unanswerable Questions

The AI chatbot properly responded to 92% of participants’ questions that have already been embedded or trained in the chatbot algorithms. However, in 31 human-chatbot interactions, the AI chatbot failed to respond or provide information for participants' questions. For example, a participant requested to interact with an actual human agent through the AI chatbot, but the AI chatbot failed to do so. Another example is that a user inquired about the existence of NGO clinics in Sepang District, Malaysia, but the AI chatbot failed to provide information.

### Chat Bubble Count

We analyzed the number of speech bubbles generated during the human-chatbot interactions. In total, the AI chatbot and participants produced 9665 and 4597 speech bubbles, respectively. On average, the AI chatbot produced 24.6 (SD 14.7) speech bubbles, ranging from 2 to 107, while users contributed an average of 11.7 (SD 8.6) speech bubbles, with a range of 1 to 71. Notably, when participants inquired about all 3 topics (HIV testing, PrEP, and mental health), the number of speech bubbles increased.

### Duration of Human-Chatbot Interaction, Contact Frequency, and Topics

The participants’ interaction time with the AI chatbot varied, ranging from less than 1 minute to 31 minutes. Among the 393 interactions, 21 (5.3%) interacted with the AI chatbot for less than 1 minute. A total of 303 (77.1%) interactions were conducted over 1 minute to 5 minutes, while 42 (10.7%) interactions took place between 6 to 10 minutes, and 27 (6.9%) interactions extended beyond 10 minutes.

Most participants (91%) interacted with the AI chatbot only once, while 24 (7.2%) engaged with the AI chatbot twice at different times during the study. Four participants (1.2%) interacted with the AI chatbot 3 times, and 2 participants (0.6%) engaged with the AI chatbot 4 times during the study. The most selected topics provided by the AI chatbot were HIV testing (79.6%), followed by PrEP (25.7%) and mental health (12.5%). In this study, 260 (66.2%) orders for HIV self-testing kits were submitted.

### Depression

Only 27 (6.9%) participants were assessed for depression, with a mean Patient Health Questionnaire-9 (PHQ-9) score of 13.4 (SD 7.8). Based on the PHQ-9 scoring system, 6 participants were identified as having mild depression, 2 as moderate, 2 as moderately severe, and 8 as severely depressed.

### Clinic Information

The AI chatbot provided participants with information on 3 types of clinics–HIV, PrEP, and mental health–including their addresses, phone numbers, and operating hours. In the 393 interactions, 93 (23.7%) participants requested clinics’ information through the AI chatbot. Of the 393 interactions, 65 (16.5%) participants inquired about PrEP clinics, 25 (5.9%) inquired about HIV testing clinics, and 16 (4.1%) requested mental health counselors’ information.

## Discussion

### Principal Findings

Our AI chatbot has the capacity to reach a large number of MSM, assisting them in receiving HIV self-testing kits and providing them with information on MSM-friendly clinics to facilitate access to services on HIV testing, PrEP, and mental health. Among all chatbot features, offering free HIV self-testing kits was the most popular, followed by inquiries about MSM-friendly clinics’ information, including addresses, phone numbers, and operating hours. The mean satisfaction score of 1.3 (SD 1.1) and mean recommendation score of 8.4 (SD 2.3) reveal participants’ favorable view of our AI chatbot. This study demonstrates that an AI chatbot embedded with culturally tailored health services and information has the potential to complement the current health care system by offering automatic and algorithm-based educational materials, consulting services, and free medical tools to facilitate and empower health care consumers. However, a significant barrier to increasing the usability of this AI chatbot is the lack of sophisticated algorithms to facilitate precise and personalized interactions with participants. For example, our AI chatbot showed limitations in addressing questions that were relevant but not directly embedded or trained in its algorithms. To integrate this AI chatbot into community and clinical settings that offer HIV prevention services to MSM in Malaysia, its algorithms must be improved by integrating more sophisticated AI algorithms and by contextualizing it for specific settings. Without robust algorithms, AI chatbots will not be able to fulfill their potential to streamline processes and improve patient care. They will only be able to undertake simple tasks, such as appointment scheduling and prescription refill reminders, but will be incapable of handling more complex tasks in real clinical settings, including symptom checking, patient triage, mental health support, and answering complex health queries [11]. Not to mention providing empathetic consultation or building trust with users. To develop sophisticated AI chatbot algorithms, emerging techniques such as preference-based fine-tuning, retrieval-augmented generation, and quantum machine learning can enable researchers to build more dynamic, context-aware, and customizable chatbots. These approaches warrant systematic testing and incorporation into health care applications.

In addition to the fundamental role of technical sophistication in supporting AI chatbots’ capacities, evidence from the literature demonstrates that researchers worldwide have reached a consensus that the primary factors influencing user engagement with AI chatbots include privacy protection, trustworthiness, and accuracy of information [3,8,11,12]. Despite the promising future of integrating large language models into chatbots as AI advances, testing the efficacy of AI chatbots in HIV prevention and addressing associated ethical and practical concerns remains inadequate [12]. So far, only a limited number of studies have been conducted to evaluate the efficacy of AI chatbots in HIV prevention. These studies are mainly focused on high-income countries and regions, such as the United States, Canada [13], and Hong Kong [14]. There is a scarcity of evidence from low-income nations [15], and very few studies have investigated the feasibility of leveraging AI chatbots to facilitate self-management among people living with HIV [13]. In addition, research comparing the effectiveness of AI chatbots with conventional digital tools, such as short message service (SMS) and mobile apps, remains limited. This gap underscores the importance of conducting this study in Malaysia, as it will provide essential user feedback to enhance our AI chatbot before future efficacy testing against other digital health interventions.

Given that the ChatGPT-4 omni (ChatGPT-4o) has robust and sophisticated algorithms capable of facilitating effective human-chatbot conversations, equipping our AI chatbot with ChatGPT’s open-source algorithms might be a solution to the concern that our AI chatbot is not precise and personalized enough for use in real-world health care settings. Therefore, in future studies, we will explore the feasibility of integrating the open-sourced ChatGPT-3 algorithms (ChatGPT-4 is not open-sourced yet) with our AI chatbot to test whether an updated version would be able to overcome this issue. While this integration might be a solution, it has significant barriers. First, the information entered on ChatGPT-3 is documented and could potentially be used for other purposes by the OpenAI Inc. or other third parties; therefore, privacy is a major concern that must be addressed by researchers and chatbot developers. To maintain the confidentiality and security of the information entered, we propose that future AI chatbots equipped with self-developed algorithms and the ChatGPT-3’s open-source algorithms should be hosted on a safe and secure server owned by a responsible stakeholder, such as a university’s cloud environment, where the evaluation of information security has been completed and ethical approval has been received. For researchers who do not have such resources, we recommend defensive measures to avoid data breaches, including backing up data locally, avoiding storage of sensitive information on the cloud, encrypting all cloud data, installing antivirus software to protect encrypted data, creating strong passwords, changing passwords routinely, and requiring at least 2 identification questions to confirm the authorized users.

Second, ChatGPT’s algorithms are used for the public, not specifically designed for HIV prevention and MSM; therefore, after upgrading the algorithms, testing the AI chatbot is a key. The focus of the chatbot testing should be on the chatbot’s capability to address contextualized problems and on customized training to improve its usability based on end-users’ feedback. The testing and training will be an interactive process, during which health scientists and chatbot engineers need to closely collaborate to tailor the AI chatbot’s programming language and codes. More importantly, researchers and engineers interested in developing AI chatbots for health purposes must identify the real-world needs in health care settings. They must create user-friendly AI chatbot solutions to meet the expectations of health care professionals and fulfill the needs of health care consumers. In this study, the frequency of unanswered questions was 31 (8%), which could potentially be addressed by improving the AI chatbot’s algorithms. Although this study used an observational design, the contact frequency of 304 (91%) indicates that most participants interacted with the AI chatbot only once. This finding suggests that long-term engagement remains a concern that should be explored and tested in future research. The HIV self-testing kit order rate of 260 (66.2%) indicates that most participants primarily used the AI chatbot to order testing kits. Since participants were able to order free HIV self-testing kits through our AI chatbot, this service appeared to be a key incentive for its use. Providing additional free services and integrating them into the AI chatbot would likely increase sustained user engagement. For future implementation of AI chatbots, the priority is to establish an effective connection between the AI chatbot and trusted health care systems to reduce stigma and discrimination toward MSM, while also offering convenient and free services.

Despite the rapid evolution of research on AI chatbots in health care, the application of AI chatbots in health care systems is still in an early stage. There is a need for clear regulations and standards governing the use of AI chatbots in health care, particularly focusing on privacy protection, accuracy of chatbot outputs, cost-effectiveness, and continuous adaptation. For example, depression is a common health concern among MSM globally, and the use of AI chatbots in providing mental health support has gained attention. In our study, the AI chatbot randomly assessed a small proportion of MSM for depression and identified some as having depression. However, the rationale and rules for the random selection of participants are unknown, and after identifying participants with depression, the next step of linking them with trusted health care systems is unclear. Future research should be conducted to address these questions.

Linking AI chatbots with health care systems to promote high-quality and easy-access health care services is the ultimate goal of our chatbot project. Given that the design of this study is centered on observing users’ interaction with the chatbot, we were unable to evaluate the impact of chatbot use on actual HIV prevention behaviors. Our next step is to conduct a randomized controlled trial to test the efficacy of the AI chatbot in promoting HIV testing in the Malaysian context. We have conducted a series of formative studies, including focus group interviews and alpha and beta tests of AI chatbots [3,6,8]. Combining prior study findings with the results of this national observational study, we aim to create an AI chatbot equipped with our own chatbot algorithms and the open-source ChatGPT-3 algorithms in the social-networking app Telegram, which is a widely accepted app for embedding an AI chatbot among MSM in Malaysia, particularly among young MSM [6]. To test the efficacy of the AI chatbot, we will compare it with an attention chatbot in a longitudinal study aimed at improving HIV testing.

### Limitations

This study has contributed important information to the usability of AI chatbots in Malaysia, but it has several limitations. First, participants self-reported whether they met the study’s inclusion criteria. Since this is a web-based national observational study, we were unable to validate all participants’ eligibility. Second, we counted the number of participants using their internet protocol (IP) address. While this method is accurate in calculating the number of participants who used the same IP address to interact with the AI chatbot, it might not capture the same participant who used different IP addresses. Therefore, the actual sample size of the participants might be less than the calculated total sample size. To address these limitations, in our future randomized controlled trial, we will assign a unique study identification number to every participant along with a unique hyperlink to access the AI chatbot. In addition, since the AI chatbot randomly assessed participants’ satisfaction and recommendation scores, this approach might have introduced selection bias, as respondents may have been more engaged or satisfied users. Finally, patients with moderate to severe depression were provided with clinical information, but whether they were linked to care or referred to psychiatrists or mental health specialists for treatment and support was not clear to our study team. Future research should include this follow-up information on participants’ engagement with clinical care.

### Conclusions

The AI chatbot demonstrated high feasibility in delivering HIV self-testing kits and providing clinical information on HIV testing, PrEP, and mental health services. To enhance its usability in community and clinical settings, the AI chatbot must offer personalized health information and precise interaction, powered by sophisticated machine learning algorithms. In addition, establishing an effective connection between the AI chatbot and health care systems to eliminate stigma and discrimination toward MSM is crucial for the future implementation of the AI chatbot.

## Abbreviations

AI: artificial intelligence
IP: internet protocol
MSM: men who have sex with men
PHQ-9: Patient Health Questionnaire-9
PrEP: pre-exposure prophylaxis
